# Phenotypic and Genetic Consequences of Protein Damage

**DOI:** 10.1371/journal.pgen.1003810

**Published:** 2013-09-19

**Authors:** Anita Krisko, Miroslav Radman

**Affiliations:** 1 Mediterranean Institute for Life Sciences, Split, Croatia; 2 INSERM U1001, Faculté de Médecine, Université R. Descartes Paris-5, Paris, France; University of Geneva Medical School, Switzerland

## Abstract

Although the genome contains all the information necessary for maintenance and perpetuation of life, it is the proteome that repairs, duplicates and expresses the genome and actually performs most cellular functions. Here we reveal strong phenotypes of physiological oxidative proteome damage at the functional and genomic levels. Genome-wide mutations rates and biosynthetic capacity were monitored in real time, in single *Escherichia coli* cells with identical levels of reactive oxygen species and oxidative DNA damage, but with different levels of irreversible oxidative proteome damage (carbonylation). Increased protein carbonylation correlates with a mutator phenotype, whereas reducing it below wild type level produces an anti-mutator phenotype identifying proteome damage as the leading cause of spontaneous mutations. Proteome oxidation elevates also UV-light induced mutagenesis and impairs cellular biosynthesis. In conclusion, protein damage reduces the efficacy and precision of vital cellular processes resulting in high mutation rates and functional degeneracy akin to cellular aging.

## Author Summary

Cellular life is maintained by the activities of proteins that, together, prevent molecular damage from occurring in the first place and repair damaged DNA, proteins and other damaged cellular components. Cellular fitness decreases due to the fact that these proteins are themselves subject to damage, leading to the progressive degeneracy of cellular functions due to diminishing protein activity and decreased precision. The ultimate liability to protein function is the irreversible oxidative protein modification, protein carbonylation. In our study, we have altered the intrinsic susceptibility of proteins to oxidative damage via alterations of translation fidelity and the accuracy of protein folding. We have found that the increased quality of proteome leads to an improved biosynthetic capacity of cells, as well as to decreased mutation rates. Since cellular aging can be defined as a progressive loss of nearly all vital cellular functions and an increase in mutation rates, this work suggests that oxidative proteome damage may be the most likely cause of aging and age-related diseases.

## Introduction

Proteome activity sustains life, whereas genome assures perpetuation of life by ongoing renewal of the proteome, granted the capacity of the proteome to repair, replicate and express the genome. Dedicated proteins determine mutation rates via the precision of the DNA replication machinery and the efficacy and precision of DNA repair systems such as DNA base pair mismatch and damage repair. Since errors in protein biosynthesis are 10^5^ times more frequent than mutations [1], it would seem reasonable to expect that these errors should, when affecting key proteins, have a cascading effect by allowing additional errors in both DNA replication, causing mutations, and protein biosynthesis, causing further errors. Leslie Orgel has proposed just such a vicious circle of biosynthetic errors as a primary cause of aging [2].

High fidelity performance of key cellular proteins is achieved through selective kinetic proofreading steps in the course of DNA, RNA and protein biosynthesis [3], [4] and by the molecular repair, error correction and maintenance (e.g., selective turnover) systems. Therefore, the quality of the proteome is expected to affect the quality of the genome as well as the catalytic activities, the precision of protein interactions and the control of gene expression. Here we investigate the effects of physiological oxidative damage, inflicted specifically to proteins, on cellular biosynthetic systems at both the genome and proteome levels. We test the prediction that proteome damage should affect cell fate - mutagenesis and survival - more than does the inflicted reparable genome damage.

Studies of induced mutagenesis typically measure DNA damage inflicted by the mutagenic agent, ignoring the fact that DNA damaging treatments also produce oxidative damage to proteins and other cellular components. Induced mutations arise by the processing of residual (unrepaired) DNA damage, therefore the efficacy of relevant repair and replication proteins should determine also the frequency of induced mutations. We have measured major oxidative damage to proteins (irreversible protein carbonylation, PC) and DNA (reparable 8-oxoguanine) and found a remarkable correlation between PC and both spontaneous and UVC light-induced mutagenesis, as well as reduced DNA repair activity.

Our results lend support to Orgel's error catastrophe hypothesis by showing that protein damage can lead to, or even directly produce, DNA mutations. However, unanticipated by Orgel is our finding that errors in protein biosynthesis and folding predispose proteins to irreversible oxidative damage that ultimately alters or destroys their function.

## Results and Discussion

### Negative correlation between cellular biosynthetic capacity and proteome oxidation

Biological effects of oxidative stress are difficult to interpret because oxidative processes mediated by reactive oxygen and nitrogen species (ROS and RNS) damage all classes of biological molecules. To study specifically the biological consequences of irreversible oxidative damage to proteins (protein carbonylation, PC), we produced changes in intracellular PC levels, at constant levels of ROS and oxidative damage to DNA. To increase or decrease the susceptibility of proteins to carbonylation, we made use of the observations that conditions increasing errors in protein biosynthesis and folding lead to increased PC [5]–[7].

We have focused on ribosome-associated chaperone, the trigger factor (Tig, a functional homologue of the eukaryotic RAC/NAC) [8], [9] as well as chaperonin GroEL/ES and chaperone DnaK/DnaJ complexes (homologues of eukaryotic Hsp60/Hsp10 and Hsp70/Hsp40 complexes, respectively). Tig acts during the nascent polypeptide synthesis, to prevent premature folding, i.e., misfolding, of proteins exiting the ribosomal tunnel. After their release from the Tig, some proteins fold without any further assistance but, depending on their size and domain complexity, populations of “client” proteins are delivered either to the GroEL/ES or to the DnaK/DnaJ complex [8], [10].

**Figure 1:**
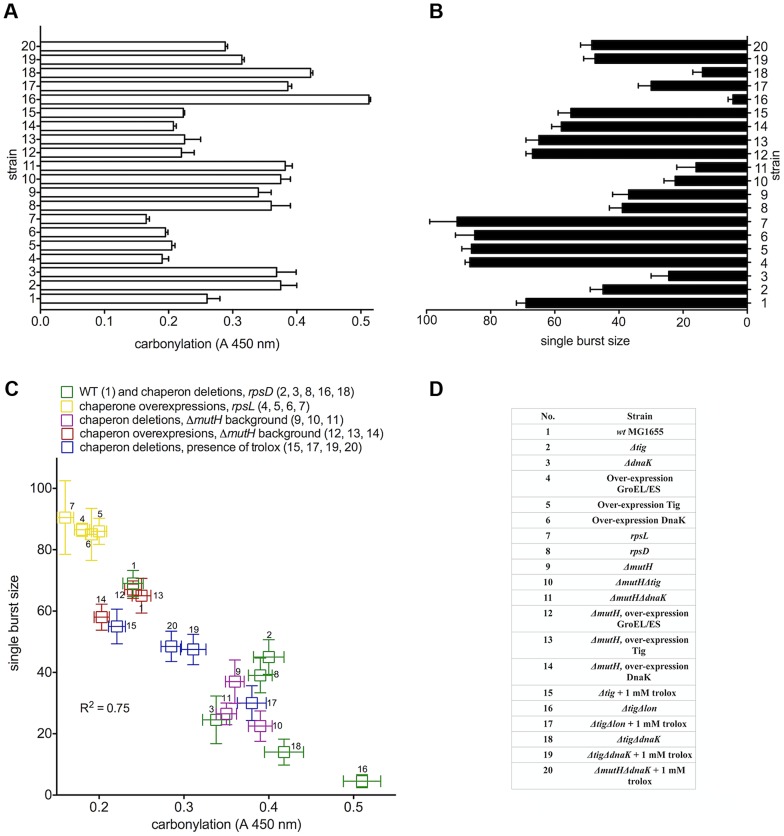
Proteome carbonylation correlates with cellular biosynthetic capacity. Exponentially growing *E.coli* strains with different levels of chaperone activity and translation errors show different levels of (a) protein carbonylation and (b) bacteriophage λ single burst size. (c) (χ) There is a negative correlation between total proteome carbonylation and biosynthetic capacity measured as single burst size of bacteriophage λ. Strain identity corresponding to the numbers is listed in (d). Results are means of 3 measurements, each in triplicate. The error bars represent the standard deviation. R^2^ value of the linear fit is indicated in panel (c). “Oe” stands for over-expression.

**Figure 2:**
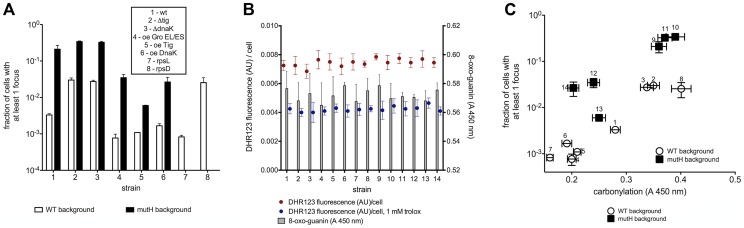
Mutation rate correlates with total proteome carbonylation. (a) The fraction of cells with a MutL-CFP focus (mutation rate) decreases with increasing chaperone activity and translation accuracy in wild type (white bars) and MutH deficient *E.coli* (black bars). (b) 8-oxo-guanine and ROS (DHR123 fluorescence) levels in each strain, in the absence and presence of 1 mM trolox, (c) Correlation between the fraction of cells with MutL-CFP foci and total proteome carbonylation. Strain identity corresponding to the numbers is listed in Figure 1D. Results are given as mean of 3 measurements, each in triplicate. Error bars represent the standard deviation. “Oe” stands for over-expression.

Using ribosomal fidelity mutants (*rpsL141* and *rpsD14*) and deletion or overexpression of three classes of chaperones (Tig, DnaK/DnaJ and GroES/EL), we show that the increase or decrease of errors in protein synthesis and folding elevates or reduces PC at constant ROS (Figure 1A and 2B). Since the accuracy in protein biosynthesis and folding affects also the saturating levels of PC upon UVC irradiation (details below), it appears that the deviation from the native structure determines protein “target size” (a subpopulation of proteins sensitive to carbonylation) for oxidative damage in a cell. These results relate to the early proposal of Kurland [11]: the decreased cellular fitness via decreased translational accuracy may be a consequence of the impact of mis-sense errors on the structures of proteins as well as on the growth of cells.

To quantify the effect of proteome oxidation on overall cellular biosynthetic capacity, we used a validated genetic method - the number of λ phages produced by individual *E. coli* cells (single burst size) - that has revealed a strong correlation between the radiation-induced PC and the single burst size [12]. Bacteriophage λ requires and diverts host cell transcription, translation and DNA replication machineries for its own reproduction. In Figure 1A and 1B, we show the cellular PC before infection (absorbance range 0.16 to 0.51) and the single burst size (range 3 to 90) after infection by a single viral particle. There is a robust negative linear correlation (R^2^ = 0.75) between the λ single burst size and physiological host cell PC (Figure 1C). The level of ROS was identical in all studied strains (Figure 2B), and the infecting viral genome was undamaged, suggesting that the large differences in λ burst size are due to large differences in the host cell PC and/or some other parallel oxidative protein damage. Moreover, the antioxidant treatment with 1 mM water-soluble vitamin E (trolox) - present only before infection - greatly improved λ production in all tested strains in proportion to the antioxidant effect on PC (Figure 1, Table S1), suggesting that PC, rather than misfolding alone, precludes biosynthesis in *E. coli*.

Double mutant Δ*tig*Δ*lon*, lacking the ribosome-associated chaperone and the main protease accumulates large amounts of carbonylated proteins, is unable to form colonies and produces only 3–5 phages per cell (Figure 1B). However, it forms colonies on plates with trolox and produces about 30 phages per cell when grown in the presence of trolox prior to infection (compare 17 and 18 in Figure 1B).

### Spontaneous mutation rates correlate with proteome oxidation

Support for the idea that proteome alterations should lead to genome alterations (mutations) came from the observation that two mutator (high mutation rate phenotype) *loci mutA and mutC* encode altered tRNAs - key elements in protein biosynthesis [13]. Here we address the question: can we detect genetic consequences of proteome damage? To determine the global genomic mutation rates in growing *E.coli* (strains listed in Figure 1D and Table S2), we used a unique method that detects every genomic mutation (each seen as a fluorescent MutL-CFP focus) emerging in the last DNA replication round - in single cells and in real time - providing directly the numbers for genome-wide mutation rates [14]. Functional MutL protein tagged with a fluorescent protein (CFP) forms persistent foci only on unrepaired mismatches that are the emerging new mutations [14]. Only the mismatch-recognizing protein MutS is required for the formation of MutL foci at unrepaired mismatches [14]. In *mutH* mutants, deficient in the late stage of mismatch repair, all replication errors remain in the newly synthesized DNA forming MutL-CFP foci such that the mutation rate corresponds to the frequency of DNA replication errors.

We found that in both wild type and in *mutH* cells, high-fidelity ribosomal mutation (*rpsL141*) and over-expression of Tig, GroEL/ES and DnaK chaperones decrease the mutation rate to create the most potent anti-mutator effect ever observed (in average, 10-fold in *mutH* and 3-fold in wild type, Figure 2A). The frequency of cells with a MutL-CFP focus is reduced from 0.3% in the wild type (corresponding to the genetic estimates [15]) to 0.09% or below. The 3-fold anti-mutator effect (p<0.0001) in the wild type is mirrored by an average of 9-fold mutator effect in strains with low-fidelity ribosome (*rpsD14*) or deletions of *tig* and *dnaK* (*groEL/ES* deletion is lethal) exhibiting around 3% of cells bearing a MutL-CFP focus (Figure 2A). The ROS levels and the levels of 8-oxoguanine, the most mutagenic oxidative DNA damage, remain equal in all strains (Figure 2B). This massive change in mutation rates is not due to changing amounts of the mismatch-binding MutS protein (Figure S1) and a standard genetic method measuring rifampicin resistant colonies [14] (Figure S2A) shows a robust correlation with the microscopic method [14], Figure S2B, Figure S3).

Damage to DNA replication, mismatch repair and other proteins assuring low mutation rates (e.g., dNTP pool biosynthesis and sanitation systems) is expected to cause elevated mutagenesis. Indeed, spontaneous mutation rates correlate well with constitutive PC (Figure 2C). Doubling PC correlates with over 100-fold increase in mutation rate.

Eliminating mismatch repair by a *mutH* gene deletion increases the frequency of cells bearing MutL-CFP foci to ∼30% (Figure 2A) – a 100-fold mutator effect. However, over-expressing Tig, GroEL/ES and DnaK chaperones in *mutH* mutant reduces emerging mutations to only 2–5% of the cells, presumably by the improved DNA replication fidelity. Deletions of *tig* and *dnaK* in the *mutH* mutant increase further the mutation rate: ∼50% of cell population bears at least one new mutation as the result of reduced replication fidelity.

### An antioxidant reduces protein oxidation and mutation rates

**Figure 3:**
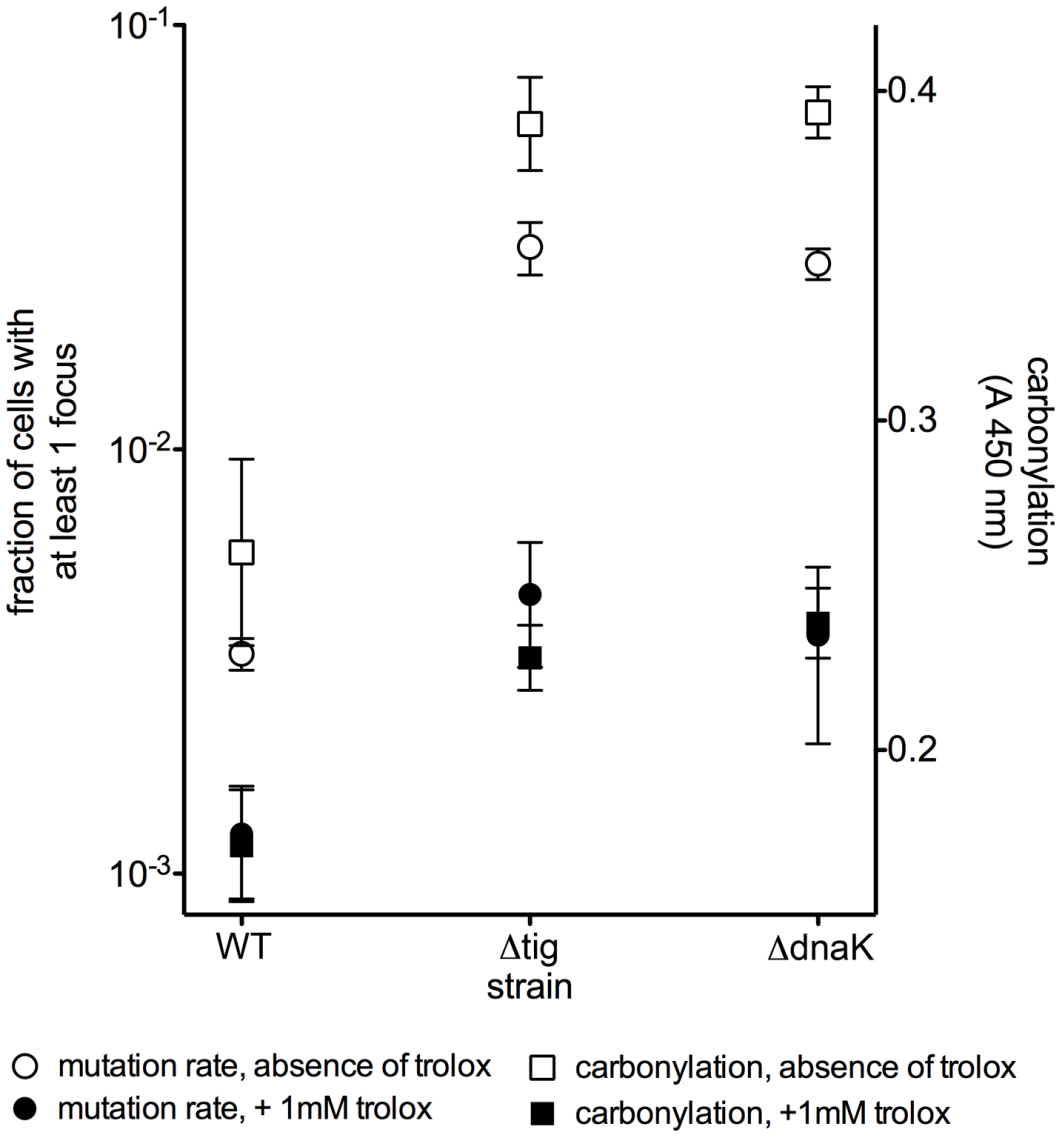
Trolox reduces the amount of protein carbonylation and the mutation rates. Parallel decrease in mutation rate (white and black circle) (fraction of cells with a MutL-CFP focus) and in constitutive protein carbonylation (white and black square) in the presence of 1 mM trolox (black symbols) relative to no trolox controls (white symbols). The results are given as mean of 3 measurements, each in triplicate. Error bars represent the standard deviation.

A key question imposes itself: are the error-bearing and misfolded proteins immediately nonfunctional or malfunctional, and their oxidation is just an epiphenomenon or a tag for proteolysis, or can the misfolded proteins still function and their activity be rescued (e.g., by chaperones) unless oxidized while in the misfolded state? Only in the latter case do we expect antioxidants to rescue protein function and only if PC (and/or some other parallel oxidative damage) is the cause of mutations do we expect a decrease in mutation rates by antioxidant treatment. We found a striking parallelism between the decrease by 1 mM trolox of both constitutive PC and mutation rates (p<0.0001) in the three strains (Figure 3). The level of suppression of intracellular ROS production (about 40%, Figure 2B) by trolox corresponds to the decrease in both PC and mutation rates (Table S1).

The results in Figures 2 and 3 identify, for the first time, the principal cause of spontaneous mutations in bacterial cells. A decrease in spontaneous mutation rates is possible only by diminishing the dominant source of mutations, if there is a single one. The anti-mutator effect of reduced PC (at constant ROS and DNA damage levels) by two means (chaperone overproduction and trolox) identifies oxidative proteome damage as the principal determinant of spontaneous mutation rates.

### Genomic polymorphism and proteome oxidation

Clearly, perturbations in the native structure, probably exposing sensitive amino acid (Lys, Arg, Pro, Thr) side chains [16], predispose proteins to oxidation. Since only a small number of carbonylated proteins can be detected on 2D western blots in *E. coli* [5], [6] and human cells [17], it seems that most native proteins have an evolved oxidation-resistant structure. Consistent observation of significantly higher PC levels in the *mutH* than in its wild type ancestor (Figure 1A) can be due to either some ongoing reversible effect of the *mutH* mutation or to the irreversible accumulation of mutations during propagation of mutH cells (at its mutator rate of 0.3 mutations per cell generation) that include oxidation-prone protein variants. Introduction of the *mutH+* copy of the gene on a plasmid (pBAD) restored the wild type mutation rate but did not decrease the PC level (not shown) suggesting that the accumulated genomic mutations (polymorphism) are the likely cause of elevated PC.

The detection of PC increase due to mutational protein polymorphism in *mutH* above the 100 times higher background of translation errors can be explained by the “monoclonality” of mutant proteins in the cell, while each random translation error affects only a single protein molecule. Given the small fraction of the *E. coli* proteome that is susceptible to carbonylation, a strong predisposition to carbonylation of few mutant high copy number proteins could increase the global PC level.

Only those polymorphic protein changes that are neutral to cell fitness, or became neutral because of the buffering effect of chaperones [18], will persist in a growing liquid culture. Indeed, the excess PC (over the wild type level) in the *mutH* mutator strain is subject to larger effects of chaperone deletions and over-expressions than in the wild type strain (Figure 1A). Hence, by measuring PC, we can detect the consequences of random protein imperfections occurring during their biosynthesis and folding, as well as of recurrent specific protein imperfections encoded by mutant genes.

### UVC light-induced mutagenesis correlates with proteome carbonylation

UV light is probably the most ubiquitous extracellular source of mutagenic activity in nature generating high amounts of ROS (Insert to Figure S4A). Highly mutagenic and cytotoxic UVC light is a model mutagen that we chose to test in strains with different chaperone activities and protein oxidation levels.

Deletions of tig or dnaK increase the maximal PC levels (Figure S4A) whereas over-expression of either Tig, DnaK or GroEL/ES chaperone complex results in lower levels of PC at saturation than in wild type cells (Figure S4B). If PC were also the key determinant of induced mutation rates, then the plateaus of PC at saturation by UVC light in different strains (Figure S4) should coincide with plateaus of UVC-induced mutation frequencies.

**Figure 4:**
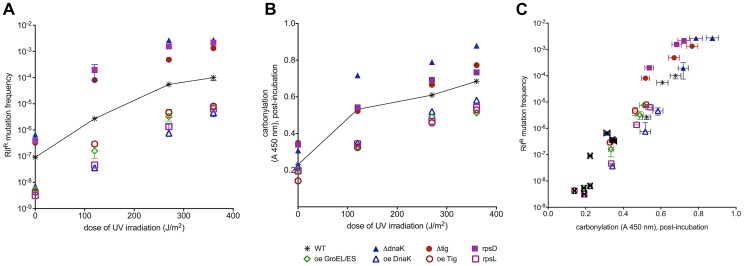
UV-induced mutation frequencies correlate with total proteome carbonylation. (a) Dose response of UVC induced mutation frequency in *E.coli* strains with increasing chaperone activity and translation accuracy. (b) Protein carbonylation at the end of the recovery period correlates with the UVC radiation dose. (c) Correlation between the UVC induced mutation frequency and protein carbonylation measured immediately after irradiation. Spontaneous mutation frequencies for each strain are labelled with an X. The results of mutation frequencies are given as median of 3 measurements, each in triplicate. Protein carbonylation measurements are presented as mean of 3 measurement, each in triplicate. Error bars represent the standard deviation. “Oe” stands for over-expression.

Since all damage-bearing mutational intermediates are not well recognized by mismatch repair proteins [14], we exploited the high potency of UVC light in inducing mutations towards rifampicin resistance (Figure 4A). UVC-induced mutation frequencies reach saturation at UVC doses that saturate also PC (between 250 and 300 J/m^2^, Figure S4). *E.coli* with low fidelity ribosomal mutation *rpsD14* and those deficient in Tig or DnaK reach a plateau at one order of magnitude higher mutation frequency (10^−3^) relative to the wild type (10^−4^). Strains bearing high ribosomal fidelity *rpsL141* mutation or over-expressing Tig, DnaK or GroEL/ES saturate at mutation frequencies of 10^−6^–10^−5^. Chaperone deletions increase the susceptibility to UVC-induced mutations at all UVC doses tested, while chaperone over-expression causes a decrease in UVC-induced mutagenesis.

Similarly to correlations of PC (Figure 4B, zero UV) with spontaneous mutation rates (Figure 4A, zero UV) in all studied strains, UVC-induced mutation frequencies extend from spontaneous mutation frequencies as parallel curves along the UVC dose range (Figure 4A). It appears that the spontaneous oxidative proteome damage sensitizes cells to UVC-mutagenesis proportionally to the level of PC before irradiation (Figure 4A). Plotting spontaneous and UVC-induced mutation frequencies versus PC in Figure 4C shows a positive correlation extending from spontaneous into UVC-induced mutation frequencies that saturate at the same UVC dose as PC in each strain.

**Figure 5:**
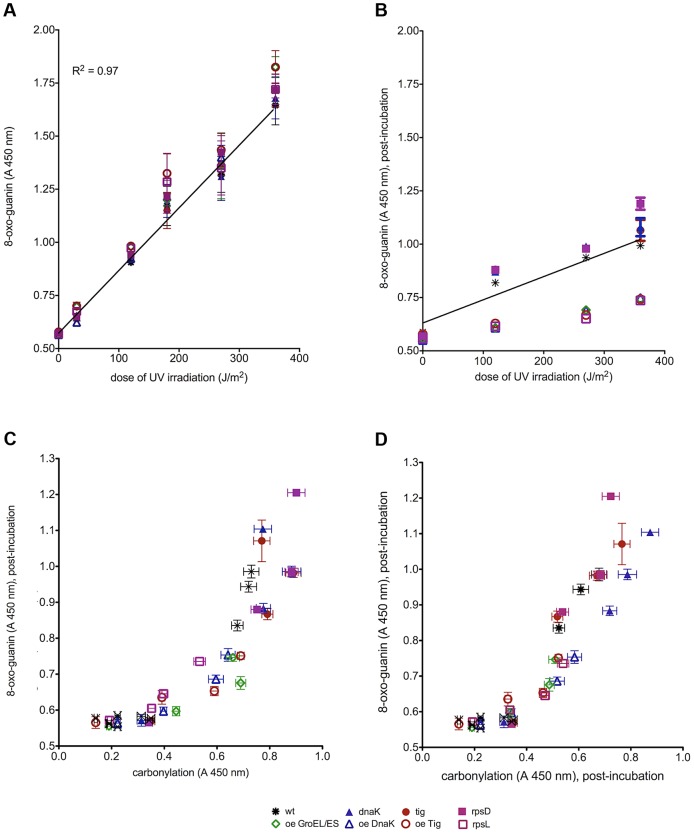
Protein damage reduces the efficiency of DNA repair. The level of 8-oxo-guanine in DNA (a) immediately after irradiation and (b) at the end of the recovery period when it increases differently with the UVC radiation depending on the fidelity of protein biosynthesis and chaperone activity. (c) 8-oxo-guanine level correlates with the protein carbonylation level immediately after irradiation. (d) 8-oxo-guanine level after post-irradiation incubation correlates with protein carbonylation. 8-oxo-guanine and protein carbonylation levels prior to irradiation are labeled with an X. The results are given as mean of 2 measurements, each in triplicate. Error bars represent the standard deviation. R^2^ value of the linear fit is indicated in panel (a). “Oe” stands for over-expression.

UVC-induced mutagenesis depends upon SOS response that induces synthesis of proteins required for the fixation of induced mutations, e.g., the RecA and DNA polymerase V [19]. The correlation between UVC-induced mutagenesis and PC does not appear to involve the SOS system because (i) the non-inducible SOS repressor mutation *lexA1* precludes UVC mutagenesis but does not alter significantly the UVC-induced PC (Figure S5) and (ii) SOS induction (measured by the expression of the *sulA* gene) in DnaK deletion or over-expression strains cannot account for the observed differences in mutagenesis (Table S3). This means that SOS system does not control induced mutagenesis by controlling protein damage and that protein damage does not control SOS induction at UVC doses tested. However, the correlation between PC and UVC mutagenesis can be accounted for by the effect of PC on residual, unrepaired, DNA damage - the substrate for UV induced mutagenesis (Figure 4 and 5). In other words, UVC-induced mutagenesis correlates with PC because PC reduces repair efficiency of mutagenic DNA damage (below).

### UVC light-induced mutagenesis versus oxidative DNA and protein damage: Protein damage reduces DNA repair

The spectra of sequenced mutations show that unrepaired DNA damage is the ultimate mutagenic intermediate in induced mutagenesis [20]. To evaluate the amount of UVC-induced oxidative DNA damage, we have measured 8-oxoguanine - one of the most mutagenic DNA lesions - in the eight strains, at several UVC doses, immediately after irradiation and after a dose-dependent post-irradiation recovery period. In contrast to both PC and mutation frequency patterns (above), (i) 8-oxoguanine level in DNA measured immediately after irradiation increases linearly with UVC dose and does not saturate in the dose range applied (Figure 5A) and (ii) the UVC-induction of 8-oxoguanine in DNA is identical for all strains regardless of their chaperone activity and translational fidelity. Clearly, the induced mutation frequencies do not correlate with the DNA damage inflicted by UVC light. After the recovery period, 8-oxoguanine level decreases in all strains as the consequence of DNA repair (Figure 5B). However, the extent of decrease varies: the *rpsL141* mutant and strains over-expressing the chaperones display a larger decrease in the 8-oxoguanine levels, i.e., more efficient repair, whereas *rpsD14* mutant and strains with chaperone deficiencies show less repair of 8-oxoguanine than the wild type (Figure 5B).

To establish the relationship between the acute protein damage (before and after exposure to UVC) and the efficacy of the post-irradiation DNA repair, we have correlated the amount of unrepaired 8-oxoguanine to the level of PC present immediately after irradiation (Figure 5C). Low initial levels of PC (*rpsL141* and chaperone over-expressions) incurred by radiation correlate with efficient removal of the 8-oxoguanine during the post-irradiation recovery. Moreover, high constitutive levels of PC (*rpsD14* and chaperone deletions) correlate with reduced post-irradiation repair of the 8-oxoguanine.

Since the unrepaired damage matters for survival and mutagenesis, we also sought for correlations between the residual 8-oxoguanine level and the residual PC level after the recovery period. Figure 5D displays the relationship between these two parameters: lower levels of the residual (post-incubation) PC correlate with more efficient repair of the UVC-induced DNA damage leaving less residual 8-oxoguanine in the DNA of the ribosomal *rpsL141* mutant and in the strains over-expressing chaperones than in wild type. High levels of residual PC (in *rpsD14* mutant and the strains with chaperone deficiencies) correlate with high levels of 8-oxoguanine remaining in the genome reflecting reduced DNA repair resulting in elevated UVC-induced mutation frequencies.

We conclude that whereas induced mutation frequencies do not correlate with the initial DNA damage inflicted by UVC, they do correlate with the residual, unrepaired, DNA damage resulting from PC related reduction in DNA repair. Hence the observed positive correlation between the cumulative (spontaneous and UVC induced) PC and induced mutagenesis.

### Conclusions and implications

We have discovered potent phenotypic effects of the damaged proteome - including mutator and anti-mutator phenotypes - by manipulating exclusively the oxidative damage to proteins. Our finding that spontaneous mutation rates and UVC light-induced mutation frequencies are determined by the level of oxidative proteome damage - rather than by incurred DNA damage - is apparently paradigm breaking. However, only unrepaired copy errors (measured here *in vivo* at single cell level) and DNA damages are mutagenic, and we show that oxidative protein damage severely limits the efficacy of DNA repair proteome.

Since reducing the physiological oxidative proteome damage by chaperone overproduction, or by antioxidant treatment, reduces spontaneous mutation rates below the wild type level, we identify oxidative proteome damage as the principal cause of spontaneous mutations in dividing bacteria. The correlation between mutation rates and protein oxidation, in the absence of mismatch repair, suggests that the precision of the DNA replication machinery is also governed by its oxidative damage. Furthermore, the frequency of induced mutations is determined by the cumulative (spontaneous and UVC light-induced) protein damage that appears as a bottleneck in the repair of UVC-inflicted DNA damage.

The accumulated oxidative protein damage, whether caused by radiation-induced ROS [12] or by increased susceptibility to ROS [5]–[7, this work], is shown here to decrease the efficacy and precision of vital biosynthetic processes (Figure 1) including DNA replication and repair (Figure 5), resulting in high mutation rates (Figures 2–4), cellular morbidity (Figure 1) and bacterial [12], [21], [22] and animal [23] cell death. Therefore the present work may be relevant to the elucidation of the still obscure basic mechanisms of natural and drug-induced killing of bacteria [24] and tumor cells [25].

Progressive loss of many vital cellular functions and increased mutation rates are the characteristics of aging human cells that accumulate PC exponentially with person's age [26] similarly to the increase in the rates of age-related diseases and death. This raises an intriguing conceptual question: Since the phenotypes of oxidative proteome damage mimic aging, is aging the phenotype of increasing proteome damage? Whereas PC is the best available biomarker of aging [26], our work suggests that PC and other protein damage might be its most likely cause. It is conceivable that the described functional and mutagenic consequences of PC apply also to human cells. In that case, the oxidative proteome damage becomes a lead candidate for a common, preventable, underlying cause of aging and age-related diseases.

## Materials and Methods

### Bacterial strains, growth conditions, and irradiation

All strains used are listed in the Table S2. They were derived from the sequenced wild-type *E. coli* MG1655 by P1 transduction and/or transformation. For all experiments, bacteria were grown in LB broth at 37°C to the exponential phase (OD600 = 0.2–0.3). It should be emphasized at this point that the double deletion mutants of E.coli were characterized by compromised growth and abundance of protein aggregates that prevented us from reliable determination of the mutation rates by the microscopic method we used.

### UVC irradiation

Bacteria were grown in LB broth at 37°C to the exponential phase (OD600 = 0.2–0.3), washed in 0.01 M MgSO_4_, and concentrated five times. All radiation experiments were performed on ice at the dose rate of 4.5 J/m^2^·s^−1^ and 254 nm. Viable cell counts were estimated by plating serial dilutions on LB plates (overnight at 37°C). In order to prevent photoreactivation, after UV treatment, all irradiated cells we kept in dark at 4°C until they were further processed.

When necessary, a post-irradiation incubation was performed with its duration depending on the irradiation dose in the following manner: 110 minutes for 120 J/m^2^, 248 minutes for 270 J/m^2^ and 330 minutes for 360 J/m^2^.

### Antioxidant treatment by Trolox

Overnight *E.coli* cultures were diluted 200-fold in LB medium supplemented with 1 mM trolox (final concentration) and grown to the mid-log phase (OD 0.2). According to the following experiment (UV irradiation, single burst size, mutation rate determination), the cells were appropriately prepared (described elsewhere in the Materials and Methods section). It is important to note that for irradiation experiments, the cells were prepared as described above, such that trolox was not present in the medium at the time of irradiation.

### Protein extracts and protein carbonylation measurement

*E. coli* cells, exponentially growing in a rich medium and irradiated, were pelleted by centrifugation immediately after irradiation and resuspended in the 10 mM PBS, pH 7.4, supplemented with a mixture of protease inhibitors containing aprotinin bestatin, leupeptin, pepstatin A, E-64 and AEBSFxHCL, EDTA-free (Pierce). Resuspended cells were frozen immediately in liquid nitrogen. Cells were broken by using a mechanical homogenizer and centrifuged 20 min at 12,000×g. Samples were then supplemented with 10 mg/100 µl lipid removal agent (Sigma 13360-U), kept 1 hr at room temperature with shaking and centrifuged 15 min at 10,000×g. The amount of protein in the supernatant was measured by the Lowry method [27]. Protein extracts diluted to 10 µg/mL were loaded into wells (Maxisorp, Nunc) and incubated over night at 4°C to allow proteins to adsorb to the surface, followed by DHR derivatization of adsorbed proteins and detection of derivatized dinitrophenol (DNP)-carbonyl by a mouse DNP specific monoclonal antibody conjugated to HRP. Subsequent incubation with enzyme substrate 3,3′,5,5′-tetramethylbenzidine resulted in a colored product that was quantified using a microplate reader with maximum absorbance at 450 nm.

### Isolation of genomic DNA and 8-oxoguanine measurement

Genomic DNA was isolated from *E.coli* strains by using Qiagen Genomic DNA Purification kit. 100 µL of genomic DNA was loaded onto the Maxisorp (Nunc) wells at the concentration of 1 µg/mL and incubated overnight at 4°C. This step was followed by an 1 hour incubation with the primary antibody mouse anti-8-deoxy-guanine and goat anti-mouse the secondary antibody conjugated to HRP. Subsequent incubation with enzyme substrate 3,3′,5,5′-tetramethylbenzidine (TMB) resulted in a colored product that was quantified using a microplate reader with maximum absorbance at 450 nm.

### Western blot determination of the MutS protein

Protein extracts were prepared as described above and a total of 10 ug of protein extracted from wild type *E. coli* MG1655 and isogenic *Δtig*, *ΔdnaK*, over-expression of GroEL/ES, Tig and DnaK strains were loaded on two SDS-PAGE gels with a 5% stacking and a 10% resolving gel. One gel was silver-stained. From the second gel, proteins were transferred onto a nitrocellulose membrane and visualized by using Amersham ECL Advance chemiluminescence detection system for imaging on autoradiographic film. Anti-MutS rabbit primary antibody (courtesy of Dr. Robert Wagner, Genecheck, USA) was used with a goat-anti-rabbit secondary antibody with horse-radish peroxidase.

### Measurement of ROS production

*E. coli* strains were labeled with 25 µM dihydrorhodamin-123 at indicated growth stage, as well as during UV irradiation. Cells were washed in minimal medium, and their fluorescence was measured (Victor 3, Perkin Elmer) with excitation at 500 nm and emission at 530 nm.

### Phage λ production: Single burst size

*E. coli* strains were grown to the mid-log phase (OD600 = 0.2) in the presence of 1% maltose (to induce high levels of λ receptor), pelleted and resuspended in 1 mL of LB broth supplemented with 30 mM MgSO_4_, 15 mM CaCl_2_, and 1% maltose (final concentrations). To count the number of phages produced per single infected cell, cells were infected at multiplicity of infection of 0.3 and diluted to obtain one cell per three tubes that were incubated in 0.4 mL LB for 60 min at 37°C. A drop of chloroform was added to help release eventual intracellular viruses. The content of each tube was mixed with 0.7% top agar supplemented with overnight culture of *E. coli* MG1655 wild type, 30 mM MgSO_4_, 15 mM CaCl_2_ and 1% maltose. Plaques were counted after overnight incubation at 37°C.

The experiment was repeated three times. Individual numbers of phage particles obtained in each of the three repetitions were pooled together and a single mean was calculated with a standard deviation, for each of the studied strains. The ranges of single burst size from each of the three experiments are summarized in Table S4.

### Microscopy: Strain construction and media

Strains studied by fluorescence microscopy are listed in Figure 1D. All strains were derived from the sequenced wild-type *E. coli* MG1655 by P1 transduction and transformation. Cells were grown on standard M9 minimal medium supplemented by 2 mM MgSO_4_, 0.003% vitamin B1, 0.001% uracil, 0.2% casamino acids, 0.01% glycerol and the required antibiotic, depending on the selection

### Live cell imaging and image analysis for counting genome-wide mutation rates

Overnight cultures of strains expressing fluorescent proteins were grown in supplemented minimal medium (see above) diluted 250-fold and re-grown to early exponential phase. Cells were concentrated and spread on agar with supplemented minimal medium, in a cavity slide to obtain a cell monolayer, as described previously [28]. The slide was mounted on Metamorph software (Universal Imaging) driven temperature-controlled (Life Imaging Services) Zeiss 200M inverted microscope. Images were recorded at 630-fold magnification using CoolSNAP HQ camera (Princeton Instruments), in phase contrast and in fluorescence (50% neutral density filter on a 100 W Fluo-Arc Hg-vapor lamp (Zeiss) regulated to 100% power) at wavelength of 500 nm during 20 seconds of exposure time. The images were analyzed by using Image J public domain software. The number of cells with MutL-CFP foci was counted manually. A total of 5000–6000 cells were examined for the wild type and MutH deficient *E.coli* in the absence of Tig and DnaK chaperones. 10.000 cells were examined for wild type and MutH deficient *E.coli* over-expressing Tig, GroEL/ES and DnaK.

### Rifampicin-resistance mutation frequencies

In order to determine the spontaneous mutation frequency, strains expressing fluorescent reporter CFP fused to wild-type MutL were grown overnight in supplemented minimal medium (see above). The overnight cultures were then diluted 10^7^-fold and grown to saturation. Dilutions of overnight cultures were plated on selective medium (LB containing 100 µg/ml rifampicin) to select rifampicin-resistant (Rif^R^) colonies, and on LB to determine the total number of colony forming units. Colonies were scored after 24 h incubation at 37°C. The median of the mutation frequency of each strain was determined from nine independent experiments, each in triplicate.

For the determination of UV-induced mutation frequencies, *E.coli* strains were grown overnight in LB medium. The overnight cultures were then diluted 200-fold until the culture reached an OD 0.2. Cells were then irradiated with 120, 270 and 360 J/m^2^. After irradiation, cells were transferred into the LB medium and incubated at 37°C with shaking (details described elsewhere in Methods). Dilutions were plated on selective medium (LB containing 100 µg/ml rifampicin) to select rifampicin-resistant (Rif^R^) colonies, and on LB to determine the total number of colony forming units. Colonies were scored after 24 h incubation at 37°C. The median of the mutation frequency of each strain was determined from three independent experiments, each in triplicate.

### β-galactosidase assay

*E.coli* strains (wild type, bearing a deletion and an overexpression of the DnaK chaperone) containing a fusion of *sulA* and *lacZ* gene (encoding β-galactosidase) were grown until OD_600_ 0.3. An aliquot of cells was exposed to chloroform in the presence of β-merkaptoethanol and incubated for 5 minutes at room temperature. Ortho-Nitrophenyl-β-galactoside (ONPG) was added (final concentration 0.6 mg/mL) and the reaction mixture was mixed by shaking for few seconds. The reaction was stopped by adding Na_2_CO_3_ (final concentration 0.5M). Tubes were centrifuged at 16,000 g for 10 minutes. OD420 was recorded in the supernatant and the units of β-galactosidase were calculated according to the formula:
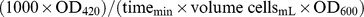



## Supporting Information

Figure S1 (a) Silver stained SDS-PAGE gel of E.coli cells, as indicated. (b)Western blot analysis of MutS levels in strains of E.coli. Oe stands for overexpression.
(TIF)

Figure S2 (a) Rif^R^ mutation frequency decreases with increasing chaperone activity in wild type (white circles) and MutH deficient (black squares) E.coli. Legend for strain numbers is listed in Figure 1D. Results are shown as mean of 9 measurements, each in triplicate. (b) Fraction of cells with at least one MutL-CFP focus displays a positive correlation with mutation frequency determined by the genetic method of Rif^R^ mutation frequency. Error bars represent the standard deviation.
(TIF)

Figure S3 Examples of MutL-CFP foci in different strains of E.coli (as indicated on the images). Arrows point into the direction of foci in the wt MG1655 panel.
(TIF)

Figure S4 Total protein carbonylation increases with UV dose and reaches saturation at (a) higher and (b) lower levels of PC for E.coli strains displaying lower and higher proteome quality, respectively. Insert to Figure S4A: Intracellular fluorescence of DHR increases linearly with dose of UVC radiation. Straight line denotes the linear fit. The results represent mean of 3 measurements, each in triplicate. Error bars represent the standard deviation.
(TIF)

Figure S5 The increase in protein carbonylation immediately after irradiation and upon post-irradiation incubation in LexA non-inducible mutant of E.coli. Error bars represent the standard deviation of three measurements, each in triplicate.
(TIFF)

Table S1 A summary of the effect of 1 mM trolox on reduction of reactive oxygen species (ROS) level, protein carbonylation (PC) amount, single burst size and the mutation rate in terms of the fraction of cells with at least one MutL-CFP focus.
(DOC)

Table S2 A complete list of E. coli strains used in this study, their genotype and source.
(DOC)

Table S3 Activity of β-galactosidase in GC4415 strain of E.coli with the deletion and overexpression of the DnaK chaperone, without and after UV induction of the SOS response.
(DOC)

Table S4 Single burst size of bacteriophage lambda. Summary of ranges in which the value of the single burst size varies over three repetitions of the same experiment.
(DOC)
