# Supplementary figures and images for: Phenotypic and Genetic Consequences of Protein Damage
Source: PLoS Genet. 2013 Sep 19;9(9):e1003810. doi: 10.1371/journal.pgen.1003810 (PMC3778015; doi:10.1371/journal.pgen.1003810)

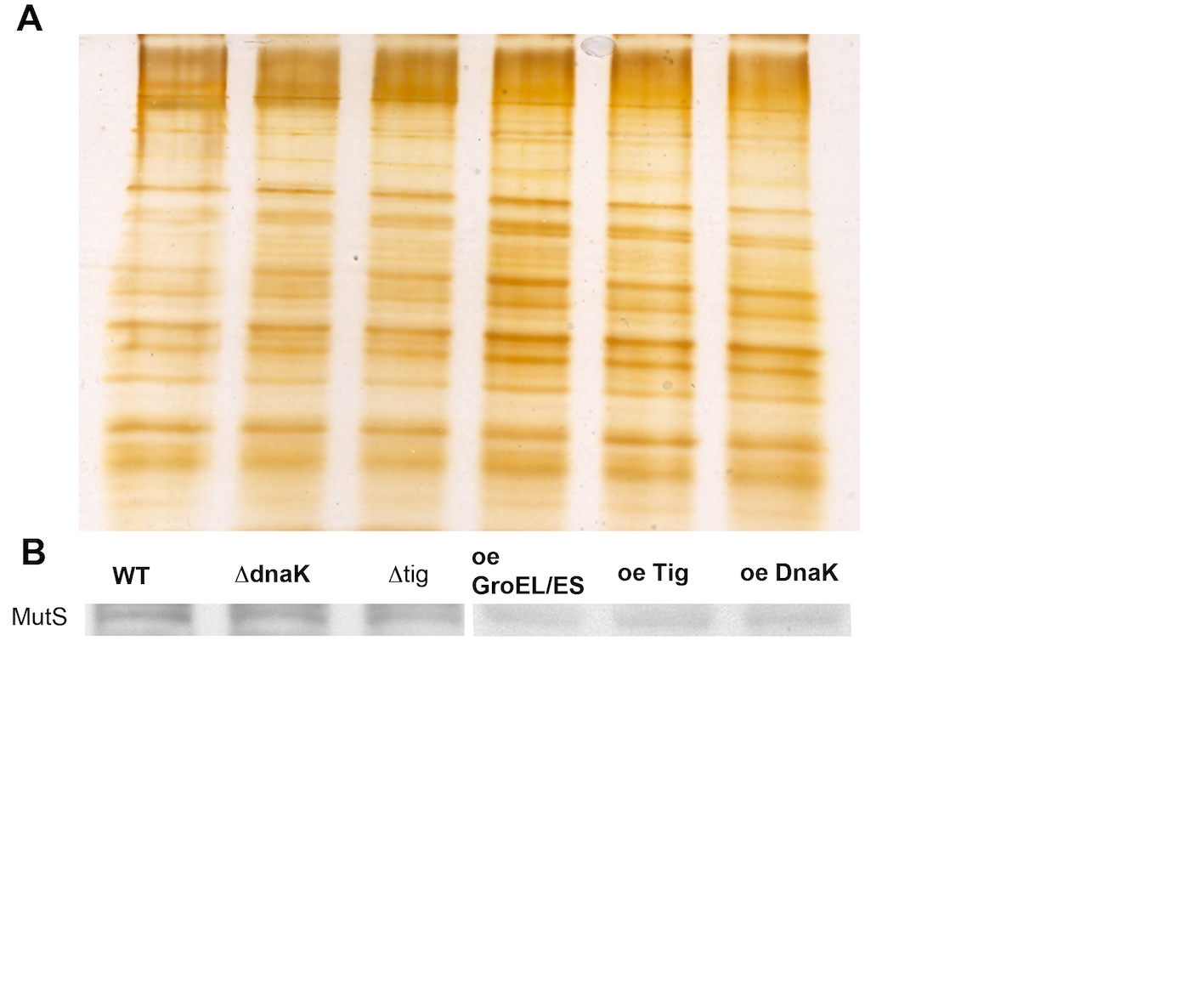

Supplement: Figure S1 — (a) Silver stained SDS-PAGE gel of E.coli cells, as indicated. (b)Western blot analysis of MutS levels in strains of E.coli. Oe stands for overexpression. (TIF) [file pgen.1003810.s001.tif]

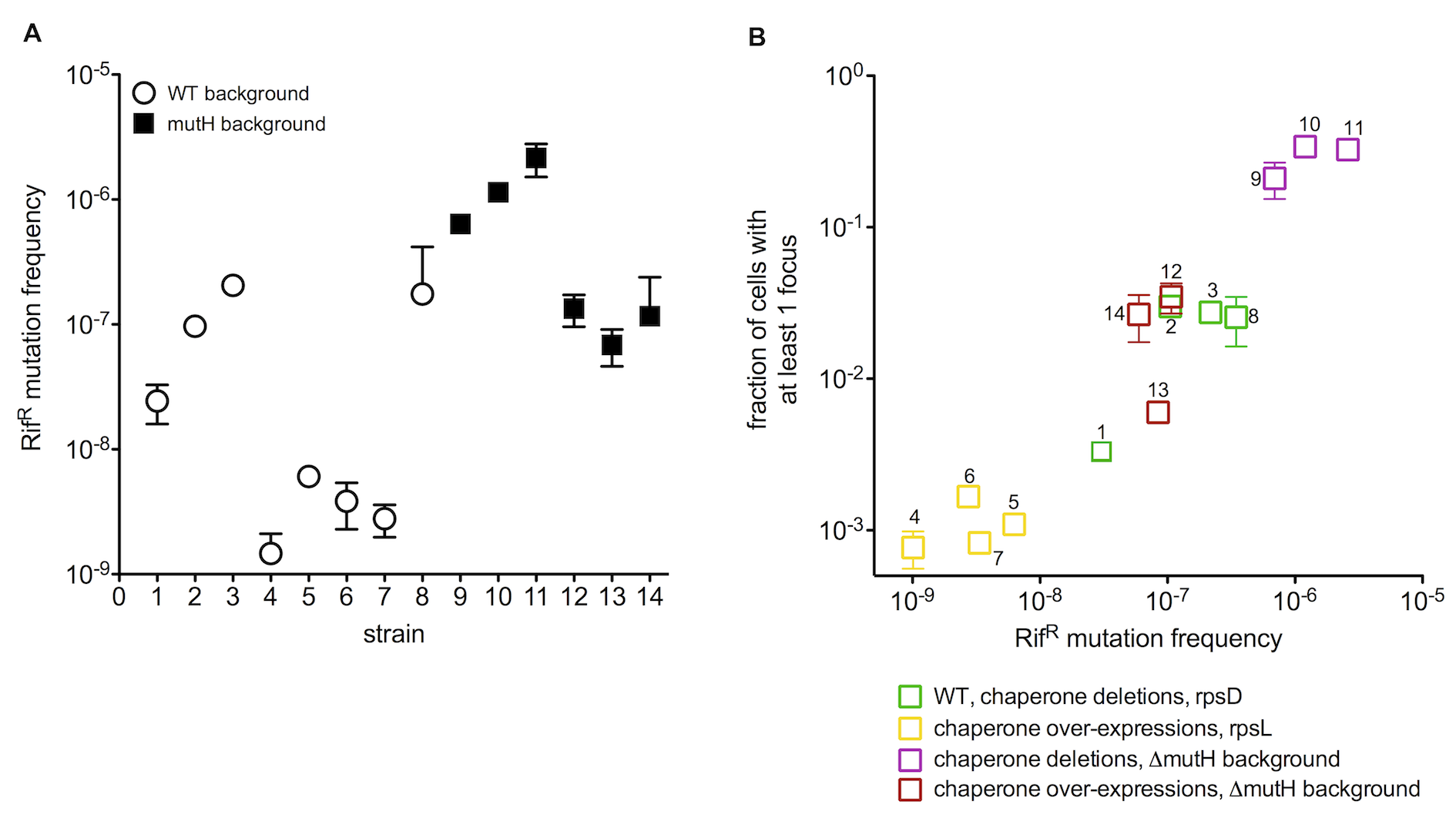

Supplement: Figure S2 — (a) RifR mutation frequency decreases with increasing chaperone activity in wild type (white circles) and MutH deficient (black squares) E.coli. Legend for strain numbers is listed in Figure 1D. Results are shown as mean of 9 measurements, each in triplicate. (b) Fraction of cells with at least one MutL-CFP focus displays a positive correlation with mutation frequency determined by the genetic method of RifR mutation frequency. Error bars represent the standard deviation. (TIF) [file pgen.1003810.s002.tif]

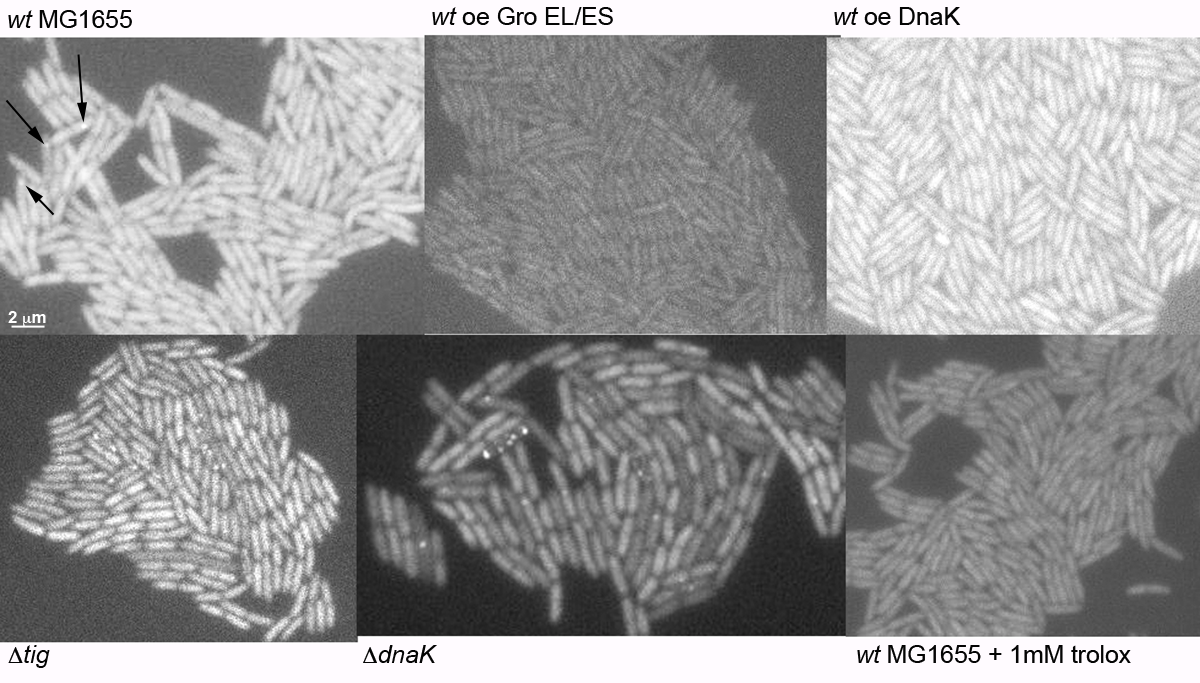

Supplement: Figure S3 — Examples of MutL-CFP foci in different strains of E.coli (as indicated on the images). Arrows point into the direction of foci in the wt MG1655 panel. (TIF) [file pgen.1003810.s003.tif]

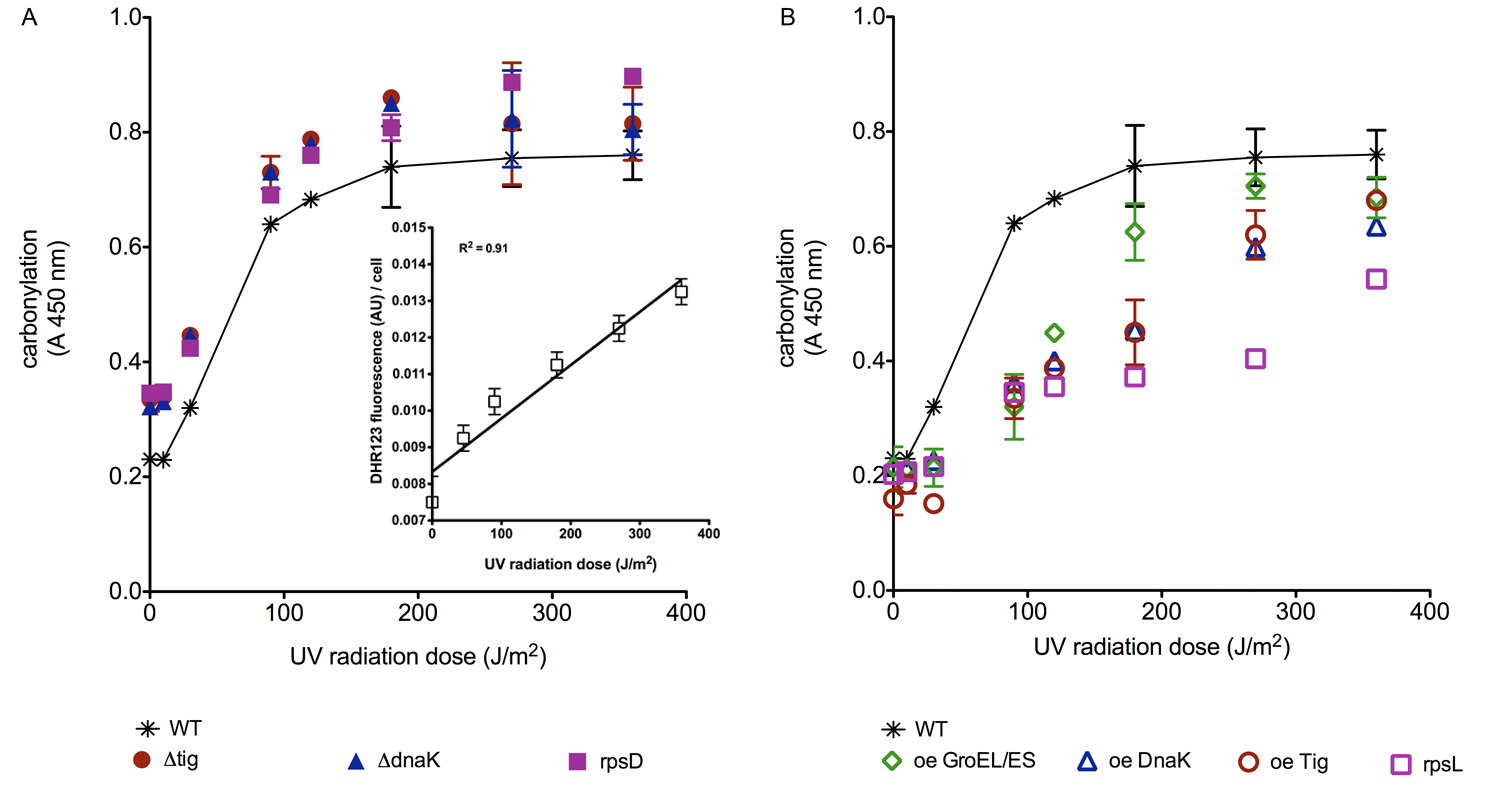

Supplement: Figure S4 — Total protein carbonylation increases with UV dose and reaches saturation at (a) higher and (b) lower levels of PC for E.coli strains displaying lower and higher proteome quality, respectively. Insert to Figure S4A: Intracellular fluorescence of DHR increases linearly with dose of UVC radiation. Straight line denotes the linear fit. The results represent mean of 3 measurements, each in triplicate. Error bars represent the standard deviation. (TIF) [file pgen.1003810.s004.tif]

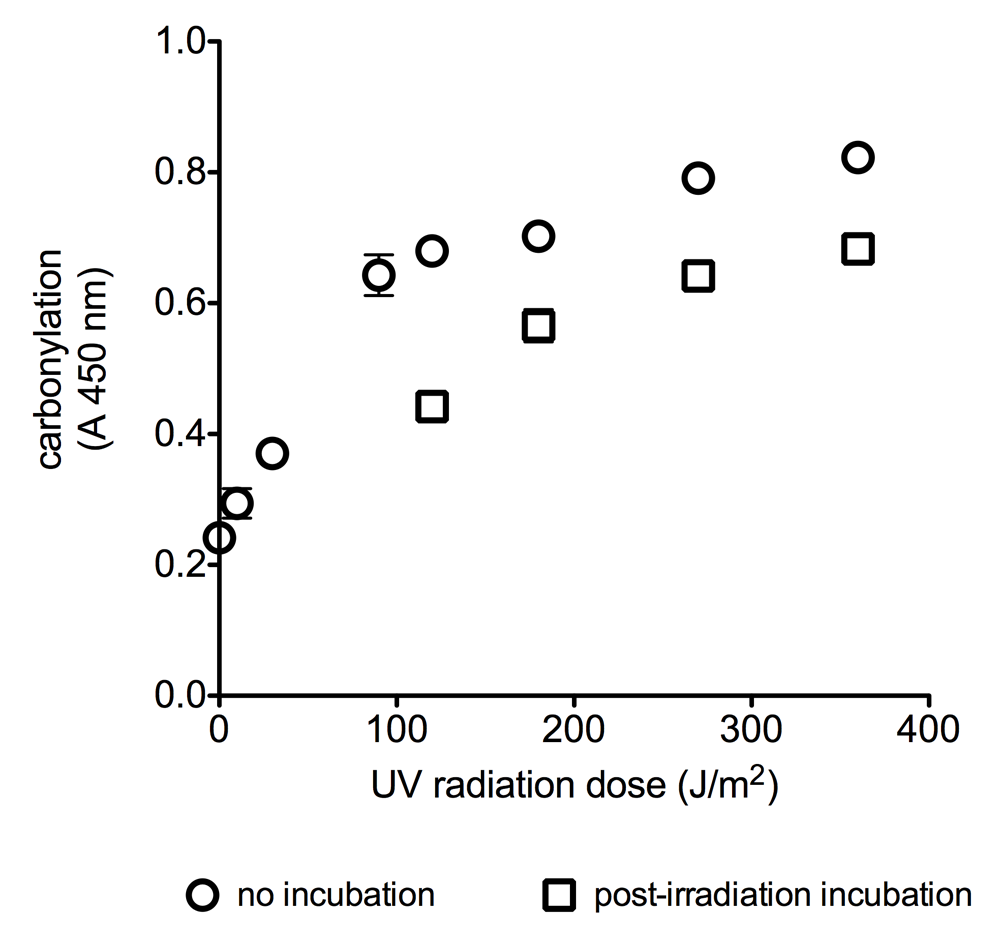

Supplement: Figure S5 — The increase in protein carbonylation immediately after irradiation and upon post-irradiation incubation in LexA non-inducible mutant of E.coli. Error bars represent the standard deviation of three measurements, each in triplicate. (TIFF) [file pgen.1003810.s005.tiff]
