# Supplementary material for: Phenotypic and Genetic Consequences of Protein Damage
Source: PLoS Genet. 2013 Sep 19;9(9):e1003810. doi: 10.1371/journal.pgen.1003810 (PMC3778015; doi:10.1371/journal.pgen.1003810)
Supplement: Table S1 — A summary of the effect of 1 mM trolox on reduction of reactive oxygen species (ROS) level, protein carbonylation (PC) amount, single burst size and the mutation rate in terms of the fraction of cells with at least one MutL-CFP focus. (DOC) [file pgen.1003810.s006.doc]

**Table S1.** A summary of the effect of 1 mM trolox on reduction of reactive oxygen species (ROS) level, protein carbonylation (PC) amount, single burst size and the mutation rate in terms of the fraction of cells with at least one MutL-CFP focus.

| 1 mM trolox effect | |  | | | |
| --- | --- | --- | --- | --- | --- |
| Strain | ROS reduction | | PC reduction | Burst size increase | Mutation rate reduction |
| wt | 0.41 | | 0.26 |  | 0.64 |
| Δtig | 0.45 | | 0.33 | 1.36 | 0.85 |
| ΔdnaK | 0.41 | | 0.25 |  | 0.87 |
