# Supplementary material for: Phenotypic and Genetic Consequences of Protein Damage
Source: PLoS Genet. 2013 Sep 19;9(9):e1003810. doi: 10.1371/journal.pgen.1003810 (PMC3778015; doi:10.1371/journal.pgen.1003810)
Supplement: Table S2 — A complete list of E. coli strains used in this study, their genotype and source. (DOC) [file pgen.1003810.s007.doc]

**Table S2.** A complete list of E. coli strains used in this study, their genotype and source.

| Strain | Genotype | Source, Reference or Construction |
| --- | --- | --- |
| MG1655 |  | Lab stock |
| JW0426 | *∆tig*::Kan | Keio collection |
| JW2703 | *∆mutS*::Kan | Keio collection |
| AK01 | As MG1655, only *∆tig*::Kan | MG1655 X P1 (JW0426) |
| AK02 | As MG1655, but pACYC184-*tig^wt^*::Tet | As MG1655 X pACYC184-Tig^wt^ |
| AF64 | MG1655 *∆lac* pBB541-g*roEL/ES*::Spec + pBB-*lacIq*:: Cam | [1] |
| AF66 | MG1655*∆lac∆lon*146::Tet | [1] |
|  | MG1655 pJK7-*dnaK*::Cam | Lab stock |
| AK03 | As MG1655, only *∆dnaK*::Kan | MG1655xP1 (JW0013) |
| IB11 | As MG1655, only *mutS*::Spec/Strep | Lab stock |
| KM55 | As MG1655, only *mutH*::Cam | Lab stock |
| JW2799 | As BW25113, only *mutH*::Kan | Lab stock |
| ME | As MG1655, only CFP-*mutL^wt^* | Lab stock |
| AK04 | As ME, only *mutH*::Cam | ME x P1 (KM55) |
| AK05 | As ME, only *mutH*::Kan | ME x P1 (JW2799) |
| AK06 | As ME, only *mutS*::Spec/Strep | ME x P1(IB11) |
| AK07 | As ME, only *mutS*::Kan | ME x P1(JW2703) |
| AK08 | As ME, only *mutH*::Kan, *mutS*::Spec/Strep | AK04 x P1(IB11) |
| AK09 | As ME, only *∆tig*::Kan | ME x P1 (ECK0430) |
| AK10 | As AK04, *∆tig*::Kan | AK07 x P1 (KM55) |
| AK11 | As AK06, only *∆tig*::Kan | AK07 x P1(IB11) |
| AK12 | As AS08, only *∆tig*::Kan | AK08 x P1(IB11) |
| AK13 | As ME, only pACYC184- *tig^wt^*::Tet | This work |
| AK14 | As AK04, only pACYC184- *tig^wt^*::Tet | This work |
| AK15 | As AK06, only pACYC184- *tig^wt^*::Tet | This work |
| AK16 | As AK08, only pACYC184- *tig^wt^*::Tet | This work |
| AK17 | As ME, only ∆lac pBB541-*groEL/ES*::Spec + pBB-*lacIq*:: Cam | This work |
| AK18 | As AK05, only *∆lac* pBB541-*groEL/ES*::Spec + pBB-*lacIq*:: Cam | This work |
| AK19 | As AK07, only *∆lac* pBB541-*groEL/ES*::Spec + pBB-*lacIq*:: Cam | This work |
| AK20 | As AK04, only *mutS*::Kan *∆lac* pBB541-*groEL/E*S::Spec | This work |
| AK21 | As ME, only *dnaK*::Kan | ME x P1(JW0013) |
| AK22 | As AK04, only *dnaK*::Kan | KM55 x P1 (JW0013) |
| AK23 | As AK06, only *dnaK*::Kan | AK05 x P1 (JW0013) |
| AK24 | As AK08, only *dnaK*::Kan | AK06 x P1 (JW0013) |
| AK25 | As ME, only pJK7-*dnaK*::Cam | This work |
| AK26 | As AK05, only pJK7-*dnaK*::Cam | This work |
| AK27 | As AK06, only pJK7-*dnaK*::Cam | This work |
| AK28 | As AK08, only pJK7-*dnaK*::Cam | This work |
| LexA1 | MG1655 *lexA1* ::Tn10 | Lab stock |
| GC4415 | MG1655 *sfiA-lacZ* ::Amp | Lab stock |
| AK29 | As GC4415, only *dnaK*::Kan | This work |
| AK30 | As GC4415, only pJK7-*dnaK*::Cam | This work |
| rpsL141 | As MG1655, carrying rpsL141 allele | [2,3] |
| rpsD14 | As MG1655, carrying rpsD14 allele | [2,3] |

Supplementary references

1. Fredriksson A, Ballesteros M, Peterson CN, Persson O, Silhavy TJ, Nyström T (2007) Decline in ribosomal fidelity contributes to the accumulation and stabilization of the master stress response regulator sigmaS upon carbon starvation. Genes Dev 21: 862-874.

2. Ballesteros M, Fredriksson A, Henriksson J, Nyström T (2001) Bacterial senescence: protein oxidation in non-proliferating cells is dictated by the accuracy of the ribosomes. EMBO J 20: 5280-5289.

3. Zaher HS, Green R. (2010) Hyperaccurate and Error-Prone Ribosomes Exploit Distinct Mechanisms during tRNA Selection. Mol Cell 39:110-120.
