# Supplementary material for: Phenotypic and Genetic Consequences of Protein Damage
Source: PLoS Genet. 2013 Sep 19;9(9):e1003810. doi: 10.1371/journal.pgen.1003810 (PMC3778015; doi:10.1371/journal.pgen.1003810)
Supplement: Table S3 — Activity of β-galactosidase in GC4415 strain of E.coli with the deletion and overexpression of the DnaK chaperone, without and after UV induction of the SOS response. (DOC) [file pgen.1003810.s008.doc]

**Table S3.** Activity of β-galactosidase in GC4415 strain of E.coli with the deletion and overexpression of the DnaK chaperone, without and after UV induction of the SOS response.

| β-galactosidase activity | |  | | | |
| --- | --- | --- | --- | --- | --- |
| Strain | spontaneous | | UV-induced, 30 minute incubation | UV-induced, 60 minute incubation | UV-induced, 120 minute incubation |
| GC4415 | 16000 ± 550 | | 19200 ± 460 | 53100 ± 510 | 114800 ± 520 |
| GC4415 ΔdnaK | 15200 ± 430 | | 26400 ± 360 | 64933 ± 480 | 166667 ± 490 |
| GC4415  oe DnaK | 16300 ± 280 | | 15300 ± 560 | 13700 ± 390 | 100800 ± 670 |
