# Supplementary material for: Phenotypic and Genetic Consequences of Protein Damage
Source: PLoS Genet. 2013 Sep 19;9(9):e1003810. doi: 10.1371/journal.pgen.1003810 (PMC3778015; doi:10.1371/journal.pgen.1003810)
Supplement: Table S4 — Single burst size of bacteriophage lambda. Summary of ranges in which the value of the single burst size varies over three repetitions of the same experiment. (DOC) [file pgen.1003810.s009.doc]

**Table S4.** Single burst size of bacteriophage lambda. Summary of ranges in which the value of the single burst size varies over three repetitions of the same experiment.

| Strain | Range of burst size, experiment 1 | Range of burst size, experiment 2 | Range of burst size, experiment 3 |
| --- | --- | --- | --- |
| wt MG 1655 | 60-72 | 66-81 | 61-69 |
| *Δtig* | 41-49 | 35-45 | 44-51 |
| *ΔdnaK* | 30-41 | 29-35 | 28-37 |
| Overexpression GroEL/ES | 86-94 | 87-101 | 84-98 |
| Overexpression Tig | 85-97 | 81-93 | 84-95 |
| Overexpression DnaK | 84-99 | 89-102 | 85-95 |
| rpsL141 | 91-102 | 82-95 | 90-101 |
| rpsD14 | 35-46 | 38-51 | 32-44 |
| *ΔmutH* | 33-41 | 35-46 | 30-39 |
| *ΔmutHΔtig* | 15-24 | 19-30 | 14-26 |
| *ΔmutHΔdnaK* | 17-25 | 16-22 | 19-26 |
| *ΔmutH,* overexpression GroEL/ES | 55-64 | 61-74 | 59-72 |
| *ΔmutH,* overexpression Tig | 59-75 | 64-73 | 58-66 |
| *ΔmutH,* overexpression DnaK | 55-64 | 52-68 | 53-64 |
| *Δtig +* 1mM trolox | 50-58 | 51-62 | 53-64 |
| *ΔtigΔlon* | 3-10 | 0-7 | 2-8 |
| *ΔtigΔlon+*1mM trolox | 19-32 | 25-36 | 22-33 |
| *ΔtigΔdnaK* | 11-22 | 14-27 | 13-26 |
| *ΔtigΔdnaK +* 1mM trolox | 41-54 | 39-49 | 35-42 |
| *ΔmutHΔlon +* 1 mM trolox | 36-41 | 33-48 | 34-46 |
